# Advancements in Viral Gene Therapy for Gaucher Disease

**DOI:** 10.3390/genes15030364

**Published:** 2024-03-15

**Authors:** Akhil Kulkarni, Tiffany Chen, Ellen Sidransky, Tae-Un Han

**Affiliations:** Section on Molecular Neurogenetics, Medical Genetics Branch, National Human Genome Research Institute, Building 35A, Room 1E623, 35A Convent Drive, MSC 3708, Bethesda, MD 20892-3708, USA; akhil.kulkarni@nih.gov (A.K.); tiffany.chen2@nih.gov (T.C.); taeun.han@nih.gov (T.-U.H.)

**Keywords:** Gaucher disease, gene therapy, vector, AAV, therapeutics, murine models

## Abstract

Gaucher disease, an autosomal recessively inherited lysosomal storage disorder, results from biallelic mutations in the *GBA1* gene resulting in deficient activity of the enzyme glucocerebrosidase. In Gaucher disease, the reduced levels and activity of glucocerebrosidase lead to a disparity in the rates of formation and breakdown of glucocerebroside and glucosylsphingosine, resulting in the accumulation of these lipid substrates in the lysosome. This gives rise to the development of Gaucher cells, engorged macrophages with a characteristic wrinkled tissue paper appearance. There are both non-neuronopathic (type 1) and neuronopathic (types 2 and 3) forms of Gaucher disease, associated with varying degrees of severity. The visceral and hematologic manifestations of Gaucher disease respond well to both enzyme replacement therapy and substrate reduction therapy. However, these therapies do not improve the neuronopathic manifestations, as they cannot cross the blood–brain barrier. There is now an established precedent for treating lysosomal storage disorders with gene therapy strategies, as many have the potential to cross into the brain. The range of the gene therapies being employed is broad, but this review aimed to discuss the progress, advances, and challenges in developing viral gene therapy as a treatment for Gaucher disease.

## 1. Introduction

### 1.1. Gaucher Disease

Gaucher disease (GD) is a very heterogeneous disorder with varying clinical manifestations and severity. Classically, GD has been classified into three types, determined by the presence (or absence) and varying degree of neurological symptoms. Type 1 (GD1), or non-neuronopathic GD, is the most common form in the Western world and is enriched in the Ashkenazi Jewish population [1]. Systemic manifestations of GD frequently include hepatosplenomegaly, thrombocytopenia, anemia, and bone involvement, although some patients exhibit few or no symptoms of the disease. Acute neuronopathic type 2 (GD2) is the most severe form of the disease, with patients exhibiting rapid onset and progression of symptoms within the first few months of life, typically succumbing to the disease between infancy and age four. Type 3 (GD3), or chronic neuronopathic GD, presents with relatively milder neurological symptoms but a wider range of phenotypes. Neurological manifestations in neuronopathic GD (nGD) can range from slowed horizontal saccadic eye movements to myoclonus, ataxia, and seizures [2,3,4]. Due to its variable manifestations and overlapping features with other diseases, the diagnosis of GD can be difficult and even missed in those with milder or later-onset presentations of the disease. Making a timely diagnosis can be critical as, in many instances, there is an optimal time to begin treatment to avoid irreversible damage [5,6]. Treatment with the current therapies can be integral for reversing or preventing hematological and visceral involvement as well as improving patients’ quality of life.

### 1.2. Approved Therapies for Gaucher Disease

Enzyme replacement therapy (ERT) is the first-line treatment for GD in patients of all ages. Current ERT for GD involves the administration of exogenously infused recombinant glucocerebrosidase to compensate for its deficiency [7]. There are three FDA-approved recombinant enzymes for Gaucher disease (imiglucerase, velaglucerase alfa, taliglucerase alfa), all of which are administered intravenously and appear to have similar efficacy [5]. Alleviation and reversal of most non-neuronopathic symptoms can be successfully achieved in most patients after an optimal dosage for the individual is determined [5,8]. Long-term cessation of enzyme replacement therapy often results in the reemergence of disease manifestations, albeit reversibly, so long as therapy is restarted in a timely fashion [8,9]. ERT is considered very safe, with reported side effects rarely warranting discontinuation of treatment [10]. However, despite its efficacy, ERT can be prohibitively expensive for many patients, limiting its availability for those who are unable to afford it, especially in developing nations [5,9,11]. Some patients cannot tolerate ERT due to poor venous access, allergy, or, in rare instances, hypersensitivity [11]. Coordinating infusions, which are typically administered in clinics, hospitals, or by home infusion nurses, can be challenging—an issue that was highlighted during the SARS-CoV-2 pandemic [9,12,13,14]. Furthermore, the recombinant enzyme is unable to cross the blood–brain barrier (BBB), rendering it ineffective for the neurological manifestations of GD observed in GD2 and GD3 [8,11,15].

For adults, including those unable to access or tolerate ERT, oral substrate reduction therapy (SRT) is a second option for the treatment of GD. While enzyme replacement therapy seeks to restore deficient glucocerebrosidase levels, substrate reduction therapy inhibits the rate of synthesis and subsequent accumulation of glycosphingolipid substrates [16]. There are currently two glucosylceramide synthase inhibitors approved as SRT for GD. Miglustat was the first SRT available [17]; it diffuses widely and rapidly into tissue and can cross the BBB, but many side effects have been reported [18]. A randomized controlled trial in 30 patients with GD3 failed to show significant neurological improvements following miglustat treatment [19]. The second SRT approved for GD, eliglustat tartrate, has greater efficacy and milder side effects than miglustat and is thus the most commonly used SRT [5,15,20]. However, eliglustat does not cross the BBB and is not approved for neuronopathic GD [21,22]. Dosing for eliglustat is dependent on the patient’s CYP26 metabolism, and the therapy is not indicated for those who are ultra-rapid CYP26 metabolizers or have any degree of hepatic impairment [5,23]. Currently, children are excluded from the therapeutic indications for SRT—an undesirable aspect given the importance of early treatment to prevent irreversible damage and developmental complications [24]. Furthermore, in contrast to ERT, the side effects observed with SRT are notably more severe, with a greater percentage of patients ending treatment as a result [5,25,26]. SRT is also still quite expensive–another barrier for patients worldwide [27]. A brain penetrant SRT, venglustat, is currently in clinical trials [28].

While current therapies for GD are highly effective and life-changing for many patients, they entail high costs and must be taken regularly for the rest of a patient’s life. Although other treatments such as bone marrow transplant are practiced in countries unable to afford ERT or SRT, these methods are considered less effective and carry a higher risk of complications [27,29]. Additionally, no current therapies are effective against neurological manifestations of GD. Thus, there is a critical, unmet need for a more affordable solution for the treatment of GD that is also capable of improving the neuronopathic aspects of the disease. Gene therapy holds potential as a curative therapy for GD that could address the shortcomings associated with current treatment options.

### 1.3. Gene Therapy

For many decades, gene therapy has been heralded as a promising therapeutic strategy for the treatment of different inherited disorders, including lysosomal storage disorders (LSDs). The ultimate goal of gene therapy is to modulate or manipulate the expression of genes in order to achieve a therapeutic effect in genetic disorders. This enables the introduction of healthy copies of a gene to replace diseased copies, the disruption of the functionality of diseased genes (through transcriptional or translational modifications), or the introduction of a novel gene for a therapeutic effect [30]. In gene therapy, the cargo (therapeutic gene) is delivered via a carrier (vector) to targeted cells. These vectors can be grouped broadly into viral and non-viral approaches. Non-viral gene therapy methods include synthetic polymers and natural polymers, which take advantage of organically derived cellular components [31,32]. Non-viral modalities offer relatively low immunogenic responses and are not limited by the size of the DNA inserts [33]. However, the poor selectivity and limited efficiency of genetic material transfer with non-viral gene therapy methods make viral gene therapy more attractive for therapeutic and clinical applications. Viral gene therapy uses vectors that are highly selective within target tissues, while also being versatile enough to differentiate amongst cells in different stages of the growth cycle [33].

Viruses that have been modified for gene therapy include retroviruses, adenoviruses, lentiviruses, and adeno-associated viruses (AAVs) [33]. Retroviruses rely on modifications of their long terminal repeats to deliver transgenes to the genome in a random integration format. However, they are associated with potential immunogenic and toxicity complications, posing safety concerns [34]. Adenoviruses were initially considered as a viable viral vector template due to their generally non-lethal properties when found in nature. However, the adenovirus may trigger a strong inflammatory response when injected into the host [35]. There are many adenovirus serotypes, but historically, group C human serotypes 2 and 5 have primarily been used in gene therapy, as they have potent transduction capacity [36]. The immunogenic risk of adenoviruses remains the primary limitation to their use as a gene therapy vector despite ongoing research [37]. Adeno-associated viruses (AAVs) offer additional advantage with respect to immunogenic protection when compared to other viral vectors [34]. Although other viral vectors have been modified to prevent infection, AAVs have an additional level of separation from infectivity that makes them attractive candidates for gene therapy. The reliance of AAVs on helper viruses precludes an adaptive response, as they have been shown to be apathogenic in humans, an important distinction to consider when comparing them to other viruses that may have previously caused infection in patients and thus trigger a strong immune response in gene therapy [38]. Twelve AAV serotypes with at least one hundred variants have been identified [39]. Adeno-associated viruses contain a small single-stranded linear genome that allows for modifications and inserts. The heterogeneity amongst these variants enables modified AAV vectors to target multiple tissue types with high infectivity. While some serotypes appear to be specific to certain tissue types, the targets of other serotypes remain unclear [40]. Currently, tissue-specific promoters in conjunction with machine learning techniques are being used to engineer more target-specific AAVs [41].

### 1.4. Benefits of AAV Vectors in Gene Therapy

The relatively small AAV genome is flanked on either side by 145 nucleotide inverted terminal repeats (ITRs), which are amenable to small therapeutic inserts of desired genes [42]. In gene therapy applications, the AAV genome is replaced with the desired foreign DNA, which is selectively designed for expression in tissues of interest. These DNA inserts encode specific transgene cassettes containing the therapeutic DNA, a regulatory sequence, a promoter, and a polyadenylation (poly(A)) signal, which ensure the appropriate mRNA processing and translation [43]. The variability amongst the many AAV serotypes further improves tissue-specific targeting and transduction into cells. The specific mechanisms for transduction into the cellular genome differ depending on the selected serotype and vector design but can include random integration of the vector genome into chromosomal regions [44] and episomal transgene expression [45]. Additionally, the safety profile of AAVs is associated with a lower immunogenic response and improved outcomes for transduction of the target gene into desired tissues and cells when compared to adenoviruses and retroviruses [34,46], suggesting that AAV is a more optimal viral gene therapy option for genetic diseases.

Many LSDs, including GD, have neurological involvement. Gene therapy offers a special advantage for neurological disorders, as many of the vectors have unique properties that allow them to permeate the BBB. AAV vectors are efficient and highly selective at transducing tissues throughout the nervous system when used in conjunction with cell-type-specific promoters and enhancers [47,48], enabling their delivery to affected brain regions. AAVs can deliver therapeutic proteins, antibodies, micro-RNAs, or precise DNA insertions and deletions to alter the genomic profile of the host cells [49]. Pre-clinical studies have suggested that direct intravenous delivery of the AAVs may be as efficacious in reversing neuronopathic phenotypes as intracerebroventricular injections exclusively targeting the central nervous system (CNS) [50]. Furthermore, several previous and ongoing clinical trials offer a cautiously optimistic view of the therapeutic potential of AAV vectors for the treatment of neurological disorders [51], supporting their safety in a clinical setting and their overall efficacy. It has also been observed that recombinant adeno-associated viral vectors are less immunogenic in all tissue types (including neuronal) than adenoviral vectors [52,53,54]. These clinical trials support the safety of AAV gene therapy in the treatment of heritable disorders and neurological disorders, making it a reasonable therapeutic approach for GD.

## 2. Current Progress in Gene Therapy for Gaucher Disease

### 2.1. Historical Overview

While there are no FDA-approved gene therapy treatments for GD presently, there has been significant interest in this field spanning several decades, including work conducted with different murine models and viral vectors. Several studies have established a historical precedent demonstrating promise for GD as a viable candidate for gene therapy. Choudary et al. [55] were among the first to explore gene therapy for GD and successfully induced the expression of human glucocerebrosidase in mammalian cells via retroviral gene transfer. However, the expressed enzyme was inactive and did not rescue GCase levels. Shortly after, the successful transplantation of transfected bone marrow cells into murine models with recovery of macrophage and central nervous system microglia was reported [56]. Schiffman et al. provided support for the efficacy of retrovirally transduced bone marrow transplantation in mice with long-term survival through repeated injections of transduced stem cells [57]. In 1997 and 1998, three separate groups performed clinical trials to assess the developing technology. Schuening et al. introduced peripheral blood repopulating cells that had been transduced with a retroviral vector to patients. However, they were unable to produce successful engraftment of transduced cells [58]. In 1998, Dunbar et al. demonstrated long-term efficacious engraftment of infused gene-marked cells, but they were unable to show improved GCase activity in patients [59]. Barranger et al. transduced modified CD34+ cells into recipients and were able to observe sustained enzyme production in one patient for at least nine months [60]. These reported levels were sufficient for the patient to be weaned off ERT, but after 27 months, enzyme levels decreased and the patient had to resume infusion therapy. Several of these early studies supported the use of viral gene therapy, while cautioning about possible safety concerns related to potential oncogenic and immunogenic side effects of the therapy [61]. The shortcomings of these clinical trials supported the need for more vigorous pre-clinical studies.

The scope of this review was focused on describing the potential efficacy of different viral vectors in vivo for gene therapy for GD. We evaluated the data presented in previous studies based on the murine models used to determine feasibility of the gene therapy treatments and their translatability, and the vector constructs and routes of administration to understand their efficacy and selectivity to certain tissue types (Table 1).

### 2.2. Murine Models

Following the unsatisfactory clinical results of ex vivo gene therapy for GD, subsequent studies turned their focus to in vivo methods. However, animal research for GD has persistently been hindered by a lack of an appropriate animal model that accurately recapitulates the phenotypes observed in humans [71,72].

The feasibility of in vivo gene therapy for GD was first demonstrated in non-Gaucher BALB/c and C57Bl/6J mouse models, which established the capability of viral vectors to produce therapeutic and supraphysiological levels of GCase in serum and Gaucher-affected tissues [62,64,65]. Marshall et al. also used an artificially induced murine model of GD by treating BALB/c mice with conduritol-β-epoxide (CBE) and glucocerebroside-containing liposomes to inhibit GCase activity and increase glycosphingolipid levels, respectively. These mice accumulated GluCer in the lysosomal compartments of liver macrophages (Kupffer cells), effectively replicating one of the biological hallmarks of GD. The group demonstrated that gene transfer-induced secreted GCase could localize to Kupffer cells despite being unmodified and that it had a longer half-life than the modified enzyme administered in ERT. Successful targeting of GCase delivered to the macrophages efficiently cleared GluCer accumulation in the liver, confirming that GD is a viable candidate for in vivo gene therapy [62].

Despite the relative ease and low cost associated with generating the CBE mouse, it is ultimately a non-genetic model that is not sufficient for evaluating the efficacy of gene therapy in patients. Furthermore, it can be a rather variable model, as symptom presentation is largely dependent on CBE dose, length of treatment, and age and strain of the mouse [73]. As such, more accurate pre-clinical studies of in vivo gene therapy for GD required a genetic murine model that displayed a consistent phenotype.

The development of the D409V/null mouse model by Xu et al. in 2003 allowed for further investigation into this therapeutic strategy [63,66,74]. While this *GBA1* variant is not commonly encountered in patients with GD, mice with the genotype D409V/null exhibited a >94% reduction in GCase activity, accumulation of glycosphingolipids, and abnormal storage cells in visceral tissues. D409V/null mice appeared to have normal behavior, fertility, and lifespans. No neurological manifestations were observed and, hence, it best modeled mild, non-neuronopathic GD [74]. However, memory deficits associated with the accumulation of α-synuclein were observed as these mice aged [75]. This finding could confound the use of this mouse line in gene therapy studies by introducing additional symptoms associated with pathologies that the vectors are not designed to treat.

Previous attempts to generate a complete knockout of *Gba1* in mice resulted in rapid neonatal death due to disruption of the skin barrier formation [76]. To circumvent this lethal skin phenotype, Enquist et al. generated the conditional Mx1-Cre^+^ *Gba1^flox/null^* knockout model, which was capable of proper fetal skin development [67]. Using the Mx1/Cre-loxP system, Cre-mediated deletion of *Gba1* exons 9–11 was postnatally induced through the administration of polyinosinic-polycytidylic acid, which activates the Mx1 promoter. GCase activity was abolished in the spleen and significantly reduced in the liver and bone marrow following exon excision. The induced *Gba1* knockout mice exhibited high levels of GluCer, splenomegaly, and microcytic anemia, and Gaucher cells were observed in hematopoietic tissue. No CNS involvement was detected due to the limited activity of the Mx1 promoter in the brain and the lifespan was normal, rendering the model most analogous to symptomatic type 1 GD [67,68].

The generation of models of the neuronopathic forms of GD has been challenging [77]. A K14-lnl knockout mouse line was developed by the Karlsson group in an effort to address the challenges previously associated with the development of an nGD murine model [78]. The loxP-neomycin disruption of *Gba1* in these mice was coupled with Cre-recombinase regulated by the keratinocyte-specific K14 promoter. This model enabled *Gba1* expression in the skin, which prevented neonatal death. At roughly 10 days of age, the mice rapidly deteriorated. They developed motor dysfunction and seizures, and neuropathologic evaluations revealed neuronal loss, microgliosis, and astrogliosis. K14-lnl mice have markedly reduced GCase activity and abnormal levels of GluCer in the brain, liver, and spleens, as well as the presence of Gaucher cells in visceral tissues. They typically succumbed within the first 2 weeks of life, thus providing a relevant, albeit short-lived, model that is representative of severe type 2 GD.

To elucidate the role of GCase-deficient microglia in the neuropathology of type 2 GD, Enquist et al. crossed their *Gba1^flox/flox^* mice with Nestin-Cre mice to generate the Nestin-flox/flox mouse [67,78]. In this model, *Gba1* was knocked out exclusively in neuronal and neuroglial cell precursors without disturbing GCase in the microglia. Nestin-flox/flox mice developed similar symptoms to the K14-lnl mice, including abnormal gait, limb rigidity, and end-stage paralysis. However, symptom onset and progression were delayed in comparison to the K14-lnl model.

Du et al. used the *Gba1^flox/flox^* mouse model to create another nGD model alongside the Nestin-flox/flox mouse [69,78]. They crossbred *Gba1^flox/flox^* with UBC-CreERT2 mice and Nestin-Cre mice to generate the *Gba1^flox/flox^*, UBC-CreERT2 and *Gba1^flox/flox^*, Nestin-Cre genotypes, respectively. Similar to the Mx1-Cre^+^ *Gba1^flox/null^* mice, *Gba1^flox/flox^*;UBC-CreERT2 is a conditional *Gba1* knockout model. In this model, Cre recombinase, bound to a mutant estrogen receptor (T2), is activated only after exposure to the chemical tamoxifen. This activation, driven by the UBC promoter, subsequently deletes *Gba1* throughout the entire body. After repeated intraperitoneal tamoxifen injections, the *Gba11^flox/flox^*;UBC-CreERT2 mice rapidly display weight loss, motor dysfunction (including abnormal gait and hyperextension of the neck), and seizures, and died within 7 days after induction. Gaucher cells were observed in the brain, liver, and spleen, and GCase activity was significantly reduced in the brain, liver, and spleen [69]. The *Gba1^flox/flox^*, UBC-CreERT2 mouse provides a viable model that mimics the systemic and CNS involvement observed in nGD, and the conditional nature of the model allows flexibility to study the effects of therapy in mice at different ages.

### 2.3. Gene Delivery Vectors and Outcomes

Glucocerebrosidase is only secreted when cells express high levels of the enzyme. Thus, constructed vectors must be able to promote production of GCase very efficiently in order to have a therapeutic benefit while maintaining a favorable safety profile [62]. To achieve such an outcome, various combinations of viral vectors, serotypes, and promoters have been tested to deliver human *GBA1* (huGBA).

#### 2.3.1. Non-AAV Gene Delivery Vector

Several non-AAV vectors have been considered for gene therapy for GD, including the adenovirus [62], retroviruses [67,79], and lentiviruses [68].

Marshall et al. created a recombinant adenovirus vector by replacing the E1 region of adenovirus serotype 2 (Ad2) with the human cytomegalovirus (CMV) immediate early promoter and enhancer [62]. A high dose of this vector in wild-type mice was able to increase GCase expression by 100 fold in the liver and 10 fold in the spleen and lungs as well as promoting secretion of GCase into serum. Testing their construct in CBE-induced murine models showed that both intravenous and intranasal delivery could produce Kupffer-targeted GCase that could reduce the accumulated GluCer in the liver. A low dose of the vector was also able to clear GluCer levels from Kupffer cells despite producing levels of GCase that were not detected in serum, likely due to the enhanced ability of adenoviruses to directly transduce Kupffer cells [62]. However, adenoviruses tend to be highly immunogenic, and the uptake of adenoviruses by Kupffer cells via the innate immune response paradoxically reduces the efficacy and longevity of the viral vector [80,81].

Enquist et al. used a retroviral vector with the spleen focus-forming virus (SFFV) enhancer-promoter to induce GCase expression in hematopoietic stem cells that were then transplanted into GD mice. This vector was capable of elevating GCase enzyme activity in the bone marrow, spleen, and liver and subsequently normalized substrate levels despite relatively low gene marking. Gaucher cells were almost fully eliminated in the treated model, whereas untreated mice continued to develop and exhibit the GD phenotype [67]. However, retroviruses possess the risk of genotoxicity, particularly when combined with a strong long terminal repeat enhancer-promoter such as SFFV and thus may not have a suitable safety profile for clinical gene therapy [82,83].

Lentiviruses such as HIV-1 possess a narrow tropism for nondividing cells such as primary T lymphocytes, CD34+ cells, dendritic cells, and macrophages, allowing for improved delivery to the desired gene therapy targets for GD [84]. Lentiviruses also demonstrate greater safety and less risk of insertional proto-oncogene activation than gammaretroviruses [85].Kim et al. evaluated the viability of an HIV-1-based lentiviral vector driven by the human elongation factor 1-α (EF-1α), which is a versatile and relatively potent promoter, particularly in hematopoietic stem cells [65,86]. EF-1α has improved stability, transgene expression, and transfection efficiency over traditional viral promoters such as CMV [87]. In C57BL6/J mice, this vector distributed widely into various cells and produced supraphysiological levels of GCase activity within various visceral tissues eight weeks after portal vein or tail vein injection. This elevated expression was consistently sustained in these tissues over the four months of the study, although no transduction was observed in the brains of the treated mice. Mice injected via portal vein exhibited greater GCase activity than mice injected via tail vein but developed mild hepatic toxicity post-injection.

Despite the demonstrated efficacy of the HIV-1-based lentivirus, the source of the vector carries the concern that the parent virus could reconstitute into a replication competent virus [88]. To ease this concern and further reduce their oncogenic risk, self-inactivating (SIN) lentiviral vectors have been developed by removing the transcriptional elements of HIV-1 [89,90]. Dahl et al. transduced bone marrow cells with SIN lentiviral vectors and transplanted them into pre-symptomatic and symptomatic Mx1-Cre^+^ *Gba1^flox/null^* mice. They compared the safety and efficacy of two SIN lentiviral vectors containing the human phosphoglycerate kinase (PGK) and CD68 promoters, respectively, against an SIN lentiviral vector with the SFFV promoter [68]. PGK is a relatively weak promoter that produces physiological rather than supraphysiological gene expression and is expressed ubiquitously, whereas CD68 is a macrophage-specific promoter [91,92]. Both vectors successfully reversed splenomegaly, elevated GCase activity, and prevented GluCer accumulation in the bone marrow, spleen, and liver when administered pre-symptomatically. In mice that had already developed symptoms, both vectors also significantly increased GCase activity. However, in contrast to the SFFV-positive control vector, which increased activity levels to 9.5 fold of wild-type levels, neither the PGK nor CD68 SIN lentiviral vectors restored GCase activity to wild-type levels. Nonetheless, the resulting activity levels were sufficient to reduce GluCer accumulation and dramatically reduce the number of Gaucher cells. Mice treated with either vector also exhibited near-normal spleen size as well as improvement in several blood parameters. All vectors displayed sustained expression up to at least 20 weeks. The SFFV promoter produced the highest levels of *GBA1* expression across all evaluated tissue types and cellular subsets, including progenitor cells. The CD68 and PGK promoters both expressed the transgene in tissue, lymphoid compartments, and granulocytes, but the CD68 promoter resulted in higher transgene expression in monocytes and macrophages. Thus, although the PGK and CD68 promoters produced less robust results than the SFFV promoter, they were still capable of preventing and reversing the GD1 phenotype without the safety risks associated with a stronger promoter like SFFV.

#### 2.3.2. AAV Gene Delivery Vectors

Adeno-associated viruses have been the primary viral vector used for in vivo gene therapy of GD given their relative safety compared to adenoviruses and lentiviruses, ability to sustain production of the transgene product, and ability to deliver the product to both dividing and nondividing cells. Although there are dozens of AAV serotypes, only a select few have been examined as vectors for GD, as they appear to have the most relevant tropisms.

The AAV2 serotype has been widely evaluated in gene therapy, and most recombinant AAVs contain the AAV2 ITR sequence [93]. This serotype exhibits preferential tropism for smooth muscle, skeletal muscle, CNS, liver, and kidney [94]. Hong et al. combined an AAV2 vector with the EF-1α promoter and delivered it via the portal or tail vein to determine its therapeutic feasibility for GD [64]. Although vector distribution, GCase expression, and GCase activity varied among tissue types, time points, and delivery methods, all treated mice exhibited significantly increased levels of GCase activity in the liver, spleen, and lung within two to six weeks after injection. However, activity began to decrease by 20 weeks after injection, with GCase activity in the lung dropping to baseline levels. Treated mice did not exhibit signs of toxicity or abnormal behavior, presenting a promising safety profile for this recombinant AAV2 vector. Despite its established efficacy, the longevity of the vector was not established, and Hong et al. posited that the AAV8 serotype would be a more optimal option for GD due to its stronger expression in liver [64].

Subsequently, Marshall et al. compared the efficacy of the AAV2 serotype to the AAV2/8 pseudotype [63]. The AAV2/8 pseudotype is a chimeric packaging plasmid in which the AAV2 capsid is removed and the AAV2 gene is fused with an AAV8 capsid. This pseudotype was designed to take advantage of the well-characterized longevity of the AAV2 serotype, as well as the immunological distinctiveness and greater liver tropism of AAV8 [95]. Marshall et al. constructed their vectors with the DC172 promoter, which produced a significantly higher GCase hepatic-restricted transgene expression than the CMV or DC190 promoters. This tissue-restricted promoter reduced the risk of off-target effects and an undesired host immune response. Both vectors produced supraphysiological levels of GCase that was secreted into the systemic circulation and normalized GluCer levels in treated mice following intravenous injection. However, the AAV2/8 vector was 50 to 100 fold more efficacious than the AAV2 vector, indicating that the AAV8 serotype is indeed more suitable for GD [63].

The same group then tested a pseudotyped AAV8 vector combined with the DC172 promoter administered intravenously in pre-symptomatic and symptomatic mice. In the pre-symptomatic mice, McEachern et al. reported supraphysiologic levels of GCase in the serum and liver and 50% of normal levels in the spleen and lungs. These findings were accompanied by normal GluCer levels and the absence of Gaucher cells, indicating that the vector was able to prevent GD pathology from developing. In older symptomatic mice, supraphysiologic levels of GCase were also attained, albeit in a dose-dependent manner. Even the lowest tested dose was sufficient to clear GluCer accumulation and storage. For both groups, the improved GCase levels were sustained for at least six months, demonstrating the long-term efficacy of the vector [66].

To measure the effectiveness of gene therapy for nGD, Massaro et al. developed an AAV9 vector with a human β-glucuronidase (GUSB) promoter [47]. AAV9 has been shown to cross the BBB and produce widespread expression in neurons as well as liver, heart, and skeletal muscle [96]. The nGD mice that were injected *in utero* did not develop behavioral symptoms, neuroinflammation, neuronal loss, or evidence of storage for up to 35 days. Longer-term analysis indicated that mice treated *in utero* appeared normal and fertile at day 70. However, by day 100, these mice performed worse on motor tasks, weighed less than wild-type littermates, and exhibited higher than normal microglial activation and astrogliosis. GCase activity and GluCer levels were similar to wild-type, although higher levels of other glycosphingolipids were detected. Thus, this AAV9 vector was effective in preventing neonatal death and delaying onset of symptoms but appeared to lose potency later in development. Massaro et al. also examined the utility of their vector when administered intravenously or intracerebroventricularly in newborns. No behavioral changes or weight loss was observed in treated nGD mice for up to at least 180 days, regardless of delivery method. Supraphysiological and physiological levels of GCase were observed in various brain regions. Evidence of neuronal loss and cortical thinning was observed in several brain regions of mice treated via IV infusion. In visceral organs, both delivery methods significantly increased GCase levels, with the IV route preventing splenomegaly and development of Gaucher cells. However, intracerebroventricular administration did not ameliorate visceral pathology despite the increased GCase. Therefore, while the AAV9 vector was able to prevent neonatal lethality in all cases, it was most effective against both neurological and visceral symptoms when delivered postnatally via IV injection [50].

Du et al. tested an AAV9 vector expressing mouse *Gba1* driven by a CMV promoter in tamoxifen-induced GD mice. The group administered the construct 15 and 30 days before tamoxifen induction to achieve peak expression prior to symptom onset as the decline following tamoxifen injection is too rapid for the vector to reach efficacious expression levels. Administration of the vector 15 days prior to tamoxifen induction did not demonstrate a therapeutic benefit, only prolonging survival by one to two days. However, intravenous vector administration 30 days prior to tamoxifen induction substantially extended lifespan to 14 times longer than untreated mice and greatly improved motor behavior. GCase activity was significantly increased in brain, liver, and spleen, and no toxic effects were detected even at the highest dose. However, the delay in transduction led the group to conclude that this vector is not a feasible approach for GD2 [69].

Du et al. also developed an AAV9 vector targeted specifically for nGD by driving *Gba1* expression with a neuron-specific Synapsin 1 (hSyn1) promoter in mice whose *Gba1* gene was only deleted in neural and glial cells. The vector was administered intraperitoneally prior to symptom onset, enabling normal weight gain and doubling the lifespan of the treated mice. GCase activity was significantly elevated in the cortex of treated nGD mice compared to untreated, although liver and lung were unaffected. Neuronal loss, astrogliosis, and microglial activation were also reduced, but not fully ameliorated. Thus, the AAV9-hSyn1 vector is capable of lessening, but not preventing, brain involvement without impacting the viscera. Furthermore, only the highest dose that was tested was efficacious, indicating that the AAV9-hSyn1 vector requires relatively higher doses, which may reduce the safety profile of this construct [69].

Massaro et al. also tested a single-stranded AAV9-hSyn1 vector containing a woodchuck hepatitis virus post-transcriptional regulatory element (WPRE) to express human *GBA1*. In brain, the vector was neuron-specific, and expression was also detected in several visceral organs, as well. The vector was administered to an nGD mouse model on the day of birth, prolonging lifespan and enabling normal weight gain without evidence of neurotoxicity. Compared to wild-type controls, treated mice exhibited similar motor behavior, glycosphingolipid levels, and levels of neuroinflammatory markers in the brain at 60 and 66 days of age. GCase activity in the brain of treated mice was around 68% of wild-type, but this difference was not significant. Unlike their AAV9-GUSB construct, Massaro et al.’s AAV9-hSyn1 vector fully prevented neuronal loss and preserved cortical thickness. The AAV9-hSyn1 vector also improved visceral pathology, preventing splenomegaly and Gaucher cell accumulation in liver, spleen, and heart. Thus, this construct showed promise for treating both neuronopathic forms of GD [70].

Several factors may have contributed to discrepant findings in the studies by Massaro et al. compared to Du et al. For example, Massaro et al. tested an 8-fold higher dose and injected their mice at a younger age [70]. Additionally, WPRE has been demonstrated to enhance transgene expression in single-stranded AAV vectors, which likely also contributed to the greater efficacy observed compared to the vector constructed by Du et al. [97].

## 3. Current Clinical Trials

Currently, there are a few active gene therapy clinical trials for the treatment of GD. The first (GALILEO-1), “A Gene Therapy Study in Patients with Gaucher Disease Type 1” (NCT05324943), conducted by Freeline Therapeutics, involves the administration of a liver-directed ssAAV to participants as a one-time intravenous infusion. The group made 37 *GBA1* AAV constructs, which, when infused in mice with RC-04-26, resulted in the robust uptake of GCase by cells in spleen, bone marrow, and lung [98]. At this time, no results have been reported from the patient trial. Another active clinical trial, “Phase 1/2 Clinical Trial of PR001 in Infants With Type 2 Gaucher Disease (PROVIDE)” (NCT04411654), is being conducted by Prevail Therapeutics and Eli Lilly & Company. This trial involves the injection of LY3884961, an AAV9 construct encoding wild-type *GBA1,* intracisternally in type 2 infants pretreated with methylprednisolone and sirolimus. Oral prednisone is taken concomitantly. The study aims to evaluate the immunogenicity of AAV9 and measures GCase in the blood and cerebrospinal fluid. No results have been shared at this time. The same companies have also initiated a phase 1/2 study in GD1, AVR-RD-02, compared to enzyme replacement therapy, for the treatment of GD1 (NCT04145037 PROCEED), with intravenous administration of their construct. Studies designed to evaluate the efficacy and safety of autologous hematopoietic stem cell (HSC) gene therapy using a lentiviral vector for GD1 and GD3 (NCT05815004) by Avrobio were recently withdrawn voluntarily and not due to safety or medical reasons. However, outcome measures from the few patients who completed the 52-week clinical trial indicated low vector copy numbers per cell, slight reduction in spleen and liver size, slight reduction in glucosylsphingosine levels, no changes in hemoglobin or platelet levels, and minimal increase in GCase enzyme activity that decreased over time.

## 4. Future Directions for Gene Therapy for Gaucher Disease

The development of gene therapy for GD has focused on adeno-associated viral vectors because of their relatively low risk of immunogenicity and stable expression of the gene target. While the stable expression of target genes in the nervous system is the most important factor for the development of AAVs to treat nGD, it is also desirable to express *GBA1* systemically to treat pathology in visceral organs. Since the combination of an AAV9 serotype vector with a constitutive promoter (such as GUSB) fulfills the criteria for neurological and visceral expression, this combination has been widely used in clinical trials of gene therapy to treat neurological disorders [99] and other LSDs [100] and in pre-clinical trials for GD [50,70]. Conversely, the ubiquitous expression of AAV9 may result in undesired off-target toxic side effects [101]. To overcome this limitation, other serotypes such as AAVrh10 have been developed. These serotypes have been shown to enhance transgene expression in the central nervous system with lower immunogenic side effects when compared to AAV9 [102]. However, further testing is required to analyze their efficacy for treating nGD.

Most AAVs used in pre-clinical trials are designed using single-stranded DNA (ssAAV) genomes. The time to peak expression for these vectors is limited, as they must first be converted to double-stranded DNA to begin gene expression, which may present challenges for successfully treating forms of GD that develop symptoms perinatally [103]. The conversion of ssAAVs to their double-stranded counterparts may also reduce the efficacy of the vector. An alternate AAV construct that has been proposed uses a self-complementary vector (scAAV), which contains an inverted dimeric repeat genome that folds into dsDNA without the aid of DNA synthesis molecules. These vectors have been notably effective at transducing multiple tissue types, including nervous tissue, and circumventing the limitations associated with the conversion of ssAAVs. Vector construct size in scAAVs must be reduced to approximately 2500 base pairs to prevent the dimeric repeats from exceeding the size limitations of the normal AAV packaging capacity (approximately 4700 nucleotides); the two halves of the scAAV are thus complementary. The transduction efficiency of scAAVs should be considered when designing vectors for future gene therapy studies in GD [103].

When developing an efficacious construct, consideration must also be given to the route of administration and timing of delivery of the vector. Intracerebroventricular injection may be the most reliable method to cross the BBB, but the procedure is risky and invasive and may lead to uneven distribution of the vector. Systemic injection is therefore safer and more predictable, but these routes typically produce lower vector expression and gene marking, which may compromise effectiveness [104].

To ensure successful clinical studies of gene therapy, more rigorous pre-clinical trials must be designed to further establish the efficacy and safety of different adeno-associated viral vectors, as well as variations in those vectors (including scAAVs and AAVrh10). Additionally, longitudinal studies are necessary to establish the longevity of adequate *GBA1* expression. This might indicate the need for additional vector injections periodically or the need to supplement other treatment modalities such as SRT or ERT.

Although AAVs do not cause significant infection without the aid of a secondary viral host, they could still trigger an unpredictable immune response for a variety of reasons. Antibodies to previous infection by AAVs or adenoviruses may conceivably diminish the response to the therapy or prevent a response altogether and could be dangerous to the patient [105]. A prior adenoviral infection with a similar serotype of adenovirus may result in a strong immune response post-treatment. This could give rise to serious complications including meningitis, encephalitis, and, in rare cases, death. The immunogenicity can also be impacted by insertional mutagenesis, which can preclude genotoxicity [106]. There are alarming reports in both mammalian models and human subjects of the genotoxicity resulting in hepatocellular carcinoma (HCC) [107], despite most cases in humans being episomal and benign. Thus, it is crucial to consider vector design with respect to possible undesirable integrations into the host genome to avoid carcinogenic outcomes [101]. One side effect that has been observed in clinical trials of AAV gene therapy in other neurological diseases is thrombotic microangiopathy, which is associated with an immune activation that gives rise to vascular pathologies and may result in ischemia of the brain and other organs [99]. This may be overcome by consideration of alternate administration routes of AAV such as intra-CSF infusions, which could reduce the availability of circulating AAV [99].

Treating GD2 is particularly challenging, as the window between time of diagnosis and irreversible neurological damage may be very small. However, newborn screening campaigns have allowed for identification of cases prior to symptom development, which may be integral to administering treatment early enough to drastically alter the disease course. It still remains possible, however, that irreversible CNS damage in GD2 has already begun prenatally, as elevated glucosylsphingosine levels have been documented during early gestation [108]. Nevertheless, we remain cautiously optimistic that viral gene therapy can be used to ameliorate the symptoms in at least some forms of neuronopathic GD. These therapies should be examined thoroughly and developed carefully to improve the outcomes for individuals with nGD.

There are some ethical concerns that persist regarding the use of gene therapy in young patients. There is still inadequate clinical data to conclude that these gene therapy modalities will completely reverse—or cure—disease progression and manifestations. As such, they may only partly treat a patient’s disease at a potentially very high cost [100]. Partial therapy in GD2 may prolong, but not prevent, the neurodegenerative course. Furthermore, patients may need to continue to receive additional costly therapies. The risk of minimal improvements to a patient’s quality of life is thus important to consider and to further examine through ongoing pre-clinical and clinical trials. However, there are indications of promising results of viral gene therapy in other LSDs [100]. We thus believe that the further development and evaluation of improved gene therapy vectors, modes of administration, and construct optimization can ultimately be of significant benefit to patients with all forms of GD.

## Figures and Tables

**Table 1:** A summary of the pre-clinical gene therapy studies for Gaucher disease.

Promoter	Construct	Serotype/Viral Vector	Mouse Model	Delivery Method	Outcome and Additional Info	Source
human cytomegalovirus (CMV) immediate early promoter and enhancer	huGBA	Ad2	Chemically induced via CBE	IV to tail vein and intranasally through the nares by inspiration	High-level expression of glucocerebrosidase in the liver (100-fold endogenous levels), spleen (10-fold endogenous levels), lungs (10-fold endogenous levels), and serum (10 fold higher) compared to wild-type.	Marshall et al., 2002 [62]
DC172 promoter (hepatic restricted)	huGBA	AAV2/8	D409V/null	IV to tail vein @ 4 weeks	Expressions of (systemic) GC in animals administered AAV2/8-DC172-hGC were 50 to 100 fold higher than from the corresponding AAV2/2 vector and remained undiminished at 4 months.	Marshall et al., 2004 [63]
human elongation factor 1-α	huGBA	AAV2	C57BL/6J	IV to tail vein @ 7 weeks	GCase activity was roughly 1.5–2 times higher than untreated mice and was maintained at least 20 weeks post-treatment.	Hong et al., 2004 [64]
human elongation factor 1-α	huGBA	Recombinant lentiviral vector	C57BL/6J	IV to tail vein @ 7 weeks	GCase activity was 1.9 times higher (in spleen, lung, heart, and kidney) than untreated mice at 8 weeks after treatment. Increased GC activity persisted over 4 months.	Kim et al., 2004 [65]
DC172	huGBA	AAV8	D409V/null	IV to tail vein @ 4 weeks and 6 months	Supraphysiologic levels of glucocerebrosidase were achieved in the serum and liver and lasted roughly 6 months after treatment.	McEachern et al., 2006 [66]
spleen focus-forming virus promoter (SFFV)	huGBA	Retroviral GC vector	*Gba1* (flox/null); MX-Cre; plpC induced GD	Transplants of transfected bone marrow cells. Resuspended BM cells were transplanted into recipient 1.5–7.5-month-old mice	Robust increase in enzyme activity in BM, spleen, and liver from all mice treated with GC vector, 5 months post-transplantation.	Enquist et al., 2006 [67]
PGK, CD68, SFFV	huGBA	SIN Lentiviral vector	*Gba1* (flox/null); MX-Cre; plpC-induced GD	Transplants of transfected bone marrow cells. Resuspended BM cells were transplanted into recipient 5–8-month-old mice	SFFV.GBA increase GCase levels ~10 fold, PGK.GBA, and CD68.GBA also significantly improved GCase levels	Dahl et al., 2015 [68]
GUSB *	huGBA	AAV9	K14-lnl	First fetal transuterine injection targeting the anterior horn of the lateral ventricle of the left hemisphere of the brain. Second intracerebroventricular injection to P0 neonates. Third superficial temporal vein IV to P0–1 neonates	IV administration improved GCase expression both neurologically and viscerally.	Massaro et al., 2018 [50]
CMV	Gba	AAV9	*Gba1*(flox/flox); UBC-creERT2 tamoxifen-induced GD	IV to tail vein injection @ 4 weeks	AAV9-CMV-Gba increased mean survival rate by 14x.	Du et al., 2019 [69]
Synapsin-1 (hSynl **)	Gba	AAV9	*Gba1*(flox/flox); Nestin-Cre	IP injection on day 5 postnatal	AAV9-SYN-Gba improved GCase activity in the brain, reducing the neurological symptoms and extending the lifespan in nGD mice.	Du et al., 2019 [69]
hSynI **	huGBA	AAV9	K14-lnl	IV to superficial temporal vein to P1 neonates	Overexpression of GCase did not promote neurotoxicity in the brain of injected animals and improved the lifespan for all treated animals.	Massaro et al., 2020 [70]

* More ubiquitous; ** drives expression in neuronal populations.
